# A novel approach to promote upper-limb motor recovery in stroke survivors using assistive myoelectric control and adaptive visual feedback in virtual reality

**DOI:** 10.3389/fbioe.2025.1628679

**Published:** 2025-11-21

**Authors:** Matteo Nocilli, Andrea d’Avella, Denise Jennifer Berger

**Affiliations:** 1 Laboratory of Neuromotor Physiology, IRCCS Fondazione Santa Lucia, Rome, Italy; 2 Department of Systems Medicine, University of Rome Tor Vergata, Rome, Italy; 3 Department of Biology, University of Rome Tor Vergata, Rome, Italy; 4 Department of Systems Medicine, Centre of Space Bio-medicine, University of Rome Tor Vergata, Rome, Italy

**Keywords:** stroke, motor rehabilitation, myoelectric control, patient-tailored assistive device, neurorehabilitation technology

## Abstract

Myoelectric control may offer an engaging and effective modality for post-stroke rehabilitation. By translating residual muscle activity into control signals for virtual or robotic interfaces, it enables patients to actively participate in therapeutic tasks, even when voluntary movement is limited. However, stroke patients often exhibit abnormal muscle activation patterns, which can limit the effectiveness of such approaches. To address this, we developed a novel assistive-adaptive algorithm designed to enhance myoelectric control by promoting the learning of more functional and physiologically plausible muscle activation patterns. The algorithm operates by projecting the patient’s instantaneous muscle activity onto reference patterns, each associated with a specific movement direction. These reference patterns are selected to minimize co-contraction while maintaining high similarity to physiological muscle patterns. The output of the projection determines the direction of the assistive force provided within a virtual isometric reaching task, while the level of assistance is modulated in real-time to progressively stimulate active participation, a key factor for promoting neuroplasticity. We evaluated the system through pilot experiments with three chronic stroke patients, focusing on changes in movement planning and accuracy and on the alignment toward physiological activation patterns. Our results revealed heterogeneous but promising trends, with three participants demonstrating improvements across multiple metrics after short exposure to the assistive algorithm. Specifically, higher similarity of muscle patterns to healthy participants was often aligned with better motor performance. These findings support the feasibility of using projection-based EMG assistance to guide patients toward more effective muscle recruitment strategies. The proposed framework establishes a foundation for future longitudinal studies aimed at testing whether such short-term adaptations can consolidate into lasting neuromuscular changes, potentially enhancing functional recovery through repeated and targeted exposure to myoelectric assistance.

## 1 Introduction

Stroke is one of the leading causes of adult disability worldwide, often resulting in persistent motor impairments that compromise independence and quality of life (Langhorne et al., 2011). Among the most affected functions, upper-limb (UL) control remains particularly difficult to recover, especially in chronic patients (Coupar et al., 2012). Despite the promise shown by intensive rehabilitation protocols (Ward et al., 2019), conventional therapies often fail to produce lasting functional gains, especially when patients are not actively engaged in the therapeutic process (Wu et al., 2016). One major limitation is their inability to modify the abnormal muscle activation patterns that commonly appear after stroke, such as co-contractions (Seo et al., 2022) and spasticity (Zhang et al., 2013), which reduce movement quality and efficiency. For this reason, there is an increasing need for innovative strategies that can both enhance motor performance and promote neuroplasticity.

Emerging rehabilitation technologies, including exoskeletons (Hohl et al., 2022), brain-machine interfaces (Ramos-Murguialday et al., 2013), and virtual reality (VR) environments (Qiu et al., 2020; Fluet et al., 2021; De Pasquale et al., 2025), have shown potential to facilitate motor recovery after stroke by enabling immersive and engaging training experiences (Vidaurre et al., 2023; Amin et al., 2024). While these technologies provide controlled environments and have shown promise in supporting motor recovery, it remains unclear which specific training components are most effective in driving long-term functional reorganization of the central nervous system (Vidaurre et al., 2023; Amin et al., 2024; d’Avella et al., 2024). One of the most important factors in UL motor recovery is the intensity of training (Chang and Kim, 2013). Robot-assisted therapies, especially those using end-effector devices, allow for high-repetitive exercises at relatively low cost (Blank et al., 2014). However, these systems often guide the movement passively, without requiring the patient to generate voluntary effort (Song et al., 2013). As passive movement does not encourage motor learning (Hogan et al., 2006), they may fail to effectively promote neural reorganization. Instead, evidence indicates that at least some level of active participation during the task plays a critical role in driving plastic changes in the brain (Marchal-Crespo and Reinkensmeyer, 2009).

Recently, a work by Nocilli et al. (2024) has begun to address this need for active involvement in patients. Specifically, chronic stroke survivors performed an isometric reaching task in which they were asked to produce force with the arm immobilized in a hand-forearm orthosis to drive a virtual cursor. Participants performed the task over multiple sessions. Because the cursor moved only when the patient generated force, the task demanded continuous voluntary effort and provided clear, real-time feedback. Their study suggested that repeated, effortful practice can steer motor coordination toward a more physiological organization (Nocilli et al., 2024).

To further support voluntary movement and ensure that the patient is actively involved in the task, robotic systems controlled using electromyographic (EMG) signals have been developed (Amin et al., 2024). These devices use surface EMG electrodes to record the patient’s muscle activity and translate it into control signals for movement execution. Despite their potential, these devices often fail to deliver functional improvements in patients with severe motor deficits. As a result, these patients often do not recover any motor function due to the lack of effective rehabilitation alternatives (Winters et al., 2015).

Recent research on myoelectric control has relied on EMG signals from the non-paretic or, more appropriately, Least Affected Limb (LAL) (Sarasola-Sanz et al., 2018) or recordings from isolated muscle pairs in the paretic, i.e., Most Affected Limb (MAL) of stroke patients (Mugler et al., 2019; Seo et al., 2022). As a result, there is a lack of studies that use the MAL while maintaining a consistent, intuitive mapping between muscle activity and force output. Furthermore, the mapping from muscle activity to force is pathological in individuals with motor impairments, as it is derived from dysfunctional muscle activities (Berger et al., 2017). Therefore, a physiological mapping is essential for effectively guiding motor re-learning during myoelectric control. In addition, as patients often struggle with this type of control, providing adaptive assistance that acts on the visual feedback to help them complete the task is crucial to maintain both motivation and voluntary participation throughout training.

In this work, we aim to provide personalized assistance using myoelectric control (Berger and d’Avella, 2024) through a predefined, physiological EMG-to-force mapping. Such a mapping, derived from healthy subjects, ensures that muscle activations are interpreted consistently with typical motor coordination, rather than reflecting abnormal (dysfunctional) muscle patterns often observed after a stroke (Cheung et al., 2012; Roh et al., 2015). Importantly, we propose to regulate the level of assistance not only to guide motor execution in a physiologically meaningful direction, but also to ensure that patients can complete the tasks, even when relying on impaired muscle activity. This approach maintains active participation, which is essential for promoting cortical reorganization and facilitating motor recovery. Over time, as performance improves, the level of assistance can be gradually reduced. This encourages patients to produce more accurate and voluntary muscle activation patterns, thereby reinforcing motor re-learning through both improved performance and sustained engagement.

Here we present the development of a myoelectric control algorithm designed to generate an assistive force (Berger and d’Avella, 2024) and the results of a pilot study involving three chronic stroke patients. A single training session showed that assisted myoelectric control produced an improvement in the endpoint precision during virtual target reaching and, in two participants, even increased the accuracy of initial motor planning. In addition, one patient changed muscle activations towards more functional ones. These early gains support the feasibility of the approach and motivate a larger, multi-session study to verify lasting clinical benefits.

In summary, we hypothesize that combining voluntary effort with feedback from corrected and successful movements in VR generated by the proposed assistive myoelectric control algorithm will promote neural plasticity and contribute to functional recovery by promoting re-learning of functional muscle patterns and suppression of abnormal ones in stroke survivors.

## 2 Materials and methods

### 2.1 Participants

Three chronic stroke survivors (age: 55.7 ± 8.1; months post-stroke: 67.7 ± 34.2; FM-UL: 38.0 ± 9.0, mean ± SD) participated in this pilot study. Data from seven healthy, right-handed individuals (mean age: 24.2 ± 2.4 years) with no history of neurological disorders were collected in our previous study (Berger et al., 2013) and were used as a healthy reference group. All participants gave written informed consent, and all procedures were conducted in conformance with the Declaration of Helsinki and were approved by the Ethical Review Board of Fondazione Santa Lucia, Rome (Italy) (CE/AG4-PROG. 222-34 and CE/PROG.899, dates of approval were 2009/03/09 and 2021/01/27, respectively).

### 2.2 Experimental setup

**FIGURE 1:**
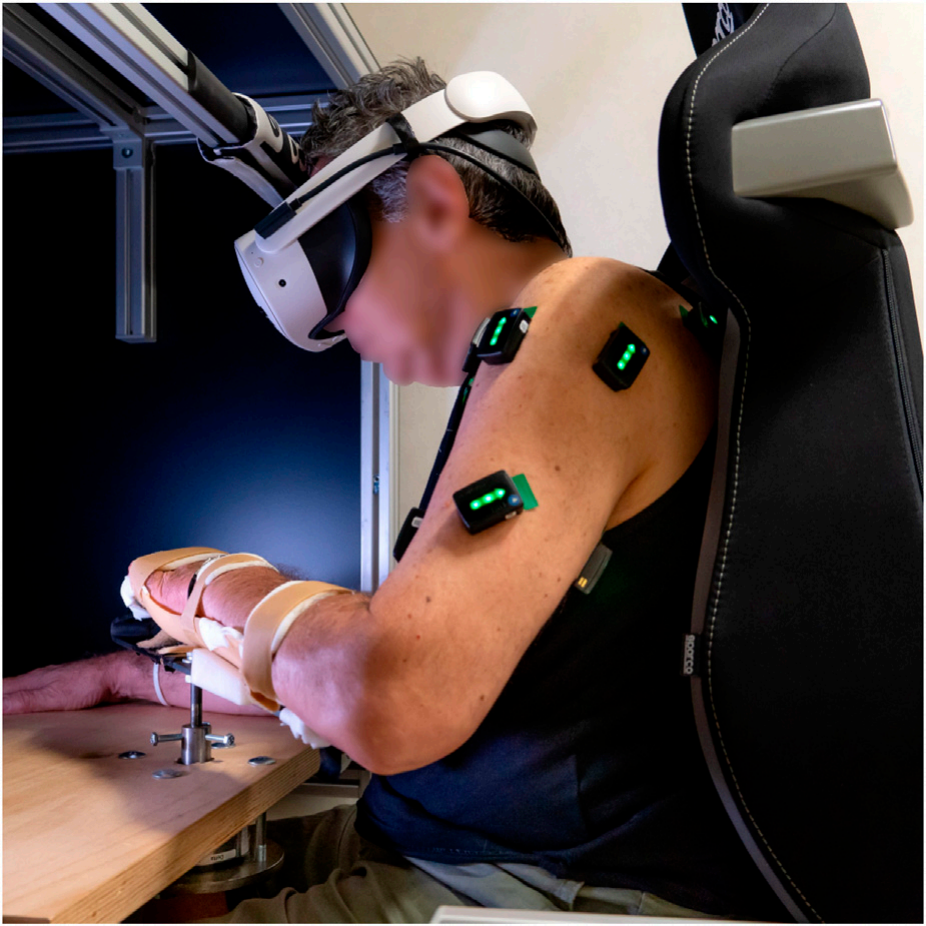
Experimental setup used by the stroke survivors, who performed the task in an immersive virtual environment through a Meta Quest 3s headset replicating the laboratory setting.

During the experiment, participants were seated comfortably on a chair in front of a table with their UL secured in a hand-forearm orthosis immobilizing the hand, wrist, and forearm (Figure 1). The base of the splint was connected through a steel bar with a cylindrical post extension to a custom-made steel socket attached to the sensitive plate of a six-axis force/torque transducer (Delta F/T Sensor; ATI Industrial Automation), mounted underneath the table surface. This sensor recorded the isometric forces and torques generated by the participant during the execution of a virtual reaching task. The task involved applying force without any physical movement of the limb, enabling the control of a virtual object through isometric contractions.

Muscle activity was measured using surface electromyographic (EMG). Signals were recorded from 13 muscles acting on the shoulder and elbow, including the brachioradialis (BracRad), short and long heads of the biceps brachii (BicShort; BicLong), lateral and long heads of the triceps brachii (TriLat; TriLong), anterior, middle, and posterior deltoid (DeltA; DeltM; DeltP), infraspinatus (InfraSp), pectoralis major (PectMaj), teres major (TerMaj), latissimus dorsi (LatDorsi), and middle trapezius (TrapMid). EMG signals of the stroke patients were acquired using wireless active bipolar electrodes (Trigno Avanti Sensor, Delsys) and bandpass filtered (30–450 Hz). In contrast, signals from healthy subjects were recorded using active bipolar electrodes (DE 2.1, Delsys), bandpass filtered (20–450 Hz), and amplified (gain 1000, Bagnoli-16; Delsys). Both force and EMG signals were digitized at a sampling rate of 1 kHz using a National Instruments PCI-6229 data acquisition board installed in a workstation computer. To extract the muscle activation envelope, EMG signals recorded from stroke patients were rectified, low-pass filtered using a fourth-order Butterworth filter with a 1.5 Hz cutoff frequency, and averaged over non-overlapping 25 ms windows while EMG signals recorded from healthy subjects were rectified, low-pass filtered using a second-order Butterworth filter with a 5 Hz cutoff frequency, and averaged over non-overlapping 10 ms windows, see (Berger et al., 2013; Berger and d’Avella, 2014) for more details. The resulting signals were baseline-corrected and normalized to the maximum value recorded from each muscle during trials in which subjects were instructed to generate maximum force in each direction.

Healthy participants performed the task with their view of the arm occluded by a 21-inch LCD monitor, which displayed the rendering of a virtual table aligned with the real workspace (Berger et al., 2013). In contrast, stroke patients wore a Meta Quest 3s headset (Meta Platforms, Inc., CA, United States), which presented them with an immersive virtual environment (Figure 1). The virtual scene, developed using the Unity software platform (Unity Technologies, CA, United States), was designed to replicate the real laboratory setting, including a virtual table matching the dimensions of the physical one. At rest, a spherical cursor was displayed at the virtual location corresponding to the center of the participant’s palm. This cursor was constrained to move within a horizontal plane and could be used to reach spherical targets appearing within the same plane, see (Berger et al., 2013) for more details.

The computation of the cursor position was handled by the data acquisition workstation running a real-time operating system (Matlab XPC). This system processes the input signals and transmits the resulting position data to a separate workstation, generating the virtual environment. Cursor dynamics were simulated in real time using an adaptive mass-spring-damper (MSD) model, following the approach described by (Park and Meek, 1995). Depending on the control modality, the virtual mass will either be driven by the actual isometric force recorded by the transducer (force control, FC) or by an estimated force derived from the participant’s EMG signals (EMG control, EC), obtained through a linear mapping of rectified and filtered EMG data (as described below in the EMG-to-force mapping section).

### 2.3 EMG-to-force mapping

EMG-to-force mapping for healthy participants: The isometric force generated at the hand is approximately a linear function of muscle activations:

f=H m

where f is the generated two-dimensional force vector, m is the 13-dimensional vector of muscle activations, and H is a matrix mapping muscle activation to force (dimensions 2 × 13). The EMG-to-force mapping ( H ) was estimated using multiple linear regression of the low-pass filtered force (second-order Butterworth; 1 Hz cutoff) and the filtered EMG signals recorded during FC (dynamic phase, i.e., time from target go until the target has been reached). In healthy individuals, we have previously demonstrated that this mapping is well-structured, allowing for effective motor control in an isometric reaching task in healthy subjects (Berger et al., 2013; Berger and d’Avella, 2014; Berger and d’Avella, 2023b; Berger and d’Avella, 2023a).

**FIGURE 2:**
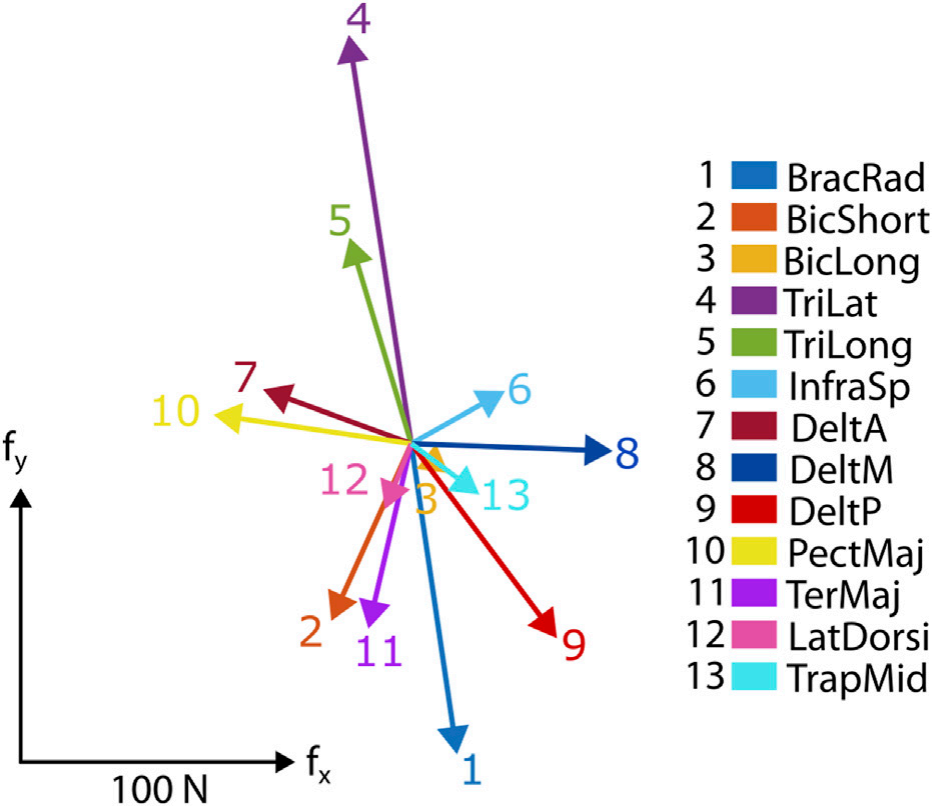
Standardized functional EMG-to-force matrix ( $H_{\text{std}}$ ). Each column of $H_{\text{std}}$ , representing the planar force generated by one muscle, is illustrated by a colored arrow (1, brachioradialis; 2, biceps brachii short head; 3, biceps brachii long head; 4, triceps brachii lateral head; 5, triceps brachii long head; 6, infraspinatus; 7, anterior deltoid; 8, middle deltoid; 9, posterior deltoid; 10, pectoralis major; 11, teres major; 12, latissimus dorsi; 13, middle trapezius).

EMG-to-force mapping for stroke patients: Myoelectric control relied on an individual-specific mapping matrix ( H ), which represents the relationship between the recorded EMG signals and the exerted force during FC blocks. While it is possible in principle to estimate such an H matrix for stroke patients as for healthy participants, we adopted a different approach. Stroke patients exhibit dysfunctional muscle activations characterized by co-contraction, defined as the synchronous activation of agonist and antagonist muscles (Silva et al., 2014), and spasticity, which refers to stiff or rigid muscles causing involuntary muscle contractions (Zhang et al., 2013). As a result, the H matrix derived from their muscle activity would also be dysfunctional, reflecting these abnormal activations. Using such a mapping matrix in myoelectric control may allow stroke patients to move the cursor correctly, but it would fail to induce proper motor re-learning. To overcome this limitation, we propose an alternative approach: replacing the patient-specific H matrix with a standardized functional one ( $H_{std}$ ) (see Figure 2). $H_{std}$ is obtained by averaging the individual H matrices of healthy participants, serving as a reference, ensuring a mapping matrix that reflects physiologically appropriate muscle activations.

### 2.4 Enhancing stroke rehabilitation through assistive myoelectric control and adaptive visual feedback

To support the re-learning of functional muscle activation patterns, we combined the use of a standardized mapping matrix, i.e., $H_{std}$ , derived from neurologically intact individuals, with an adaptive assistive mechanism for myoelectric control implemented in a virtual reality environment. The idea is to provide patients with a training context that not only facilitates task execution but also encourages correction of dysfunctional motor strategies through continuous visual feedback. In this way, the system provides salient visual feedback on the quality of motor output and may thus help patients to improve their neuromuscular control. This approach is grounded in motor learning theory, where visual feedback (Krakauer et al., 2019) and error-based correction (Patton et al., 2006; Reisman et al., 2013) are essential to adaptation. However, rather than relying on error amplification, which may overwhelm or discourage the user (Sharp et al., 2011; Marchal-Crespo et al., 2017), we propose supporting patients’ voluntary movements within the EMG-controlled virtual reality environment by correcting, in real time, the effects of pathological muscle activations in the MAL. By reinforcing the components of functional activation patterns during task execution, this strategy is expected to guide patients toward healthier motor behaviors. We thus aim at promoting neural plasticity and functional recovery by combining voluntary effort with feedback from corrected and successful movements in VR. A central element of our approach is the ability to change visual feedback in real time based on the patient’s muscle activity. This is made possible by using a virtual reality environment, where the motion of a virtual cursor is controlled by EMG signals. When a patient’s muscle activation deviates from a healthy pattern, the system adjusts the virtual movement to correct for this, guiding the user toward a more functional strategy. This process allows us to create a form of assistance that is tailored to the individual. Instead of forcing the patient to follow a fixed path, we modify the effect of their muscle activity so that the movement remains goal-directed, even if the underlying activation is impaired. As a result, patients receive feedback that is based on their own EMG signals, helping them to better understand and gradually improve how to control their muscles. This is different from passive approaches, where the limb is simply moved by the system and the patient is not actively involved. In contrast, our method requires the patient to make voluntary efforts, which are essential for promoting brain plasticity and motor recovery. As patients get better, the level of assistance is progressively reduced. This keeps the task challenging but achievable, helping to maintain motivation and encouraging continuous improvement. With time, this feedback-based training may replace abnormal muscle patterns with healthier ones (Berger and d’Avella, 2024).

### 2.5 Assistive-adaptive algorithm during myoelectric control to restore motor function

**FIGURE 3:**
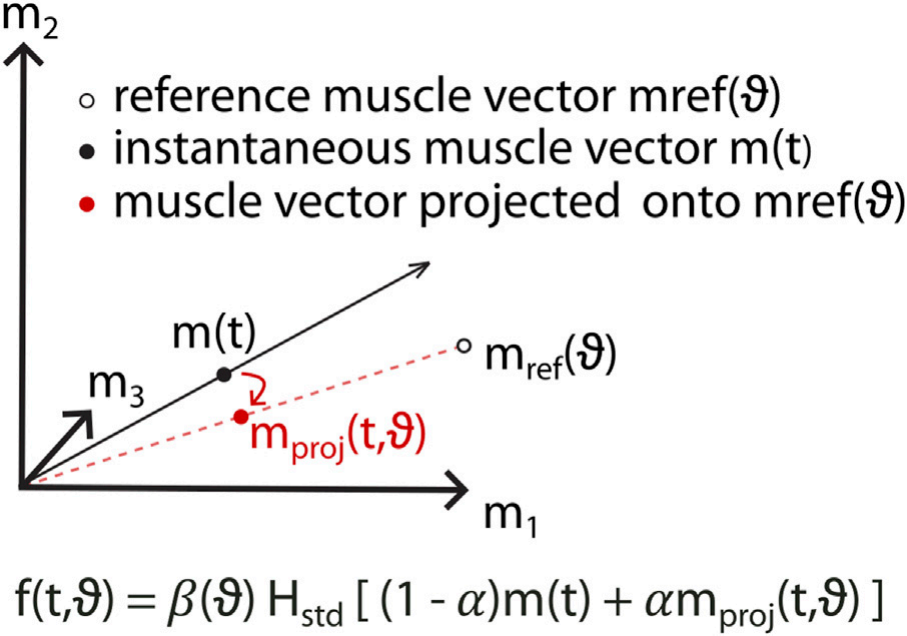
Assistive-adaptive algorithm for myoelectric control. To assist the movement toward a specific direction ( ϑ ), a target-specific reference muscle vector, derived from healthy subjects, is computed ( $m_{ref}\left(\vartheta \right)$ ) and used to project the instantaneous muscle activity vector ( $m\left(t\right)$ ) onto its direction in muscle space ( $m_{proj}\left(\vartheta \right)=P\left(\vartheta \right)\cdot m$ ). The level of assistance can be regulated by adjusting the parameter α, ranging from 1 (full assistance) to 0 (no assistance). Additional scaling in amplitude specific for each target-direction is added to enhance the visual feedback ( $\beta \left(\vartheta \right)$ ).

Based on these concepts, we developed an assistive-adaptive algorithm designed to enhance movement execution by projecting in real-time the recorded muscle activations m onto a reference muscle pattern $m_{ref}$ for a specific target direction ( ϑ ) (Figure 3):

$$m_{proj}\left(\vartheta \right)=P\left(\vartheta \right)\cdot m,\text{with }P\left(\vartheta \right)=\frac{m_{ref}\left(\vartheta \right)\cdot m_{ref}^{T}\left(\vartheta \right)}{{\left\|m_{ref}\left(\vartheta \right)\right\|}^{2}}$$

**FIGURE 4:**
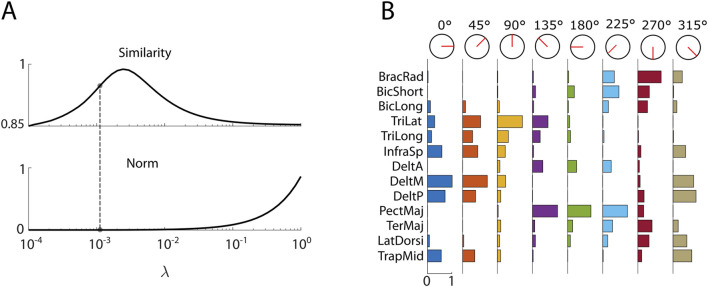
**(A)** Selection of the λ parameter used in the selection of the optimized reference pattern ( $m_{ref}$ ). The upper panel shows how the averaged similarity across target-directions between $m_{ref}$ and the non-optimized median activation pattern ( $m_{med}$ ) varies as a function of λ. The lower panel illustrates the corresponding change in the norm of $m_{ref}$ . The selected optimal lambda value is $1.2\cdot 10^{-4}$ . **(B)** Optimized reference muscle activation patterns ( $m_{ref}$ ) computed for each of the eight reaching directions. Each column represents a target direction, and the bars reflect the contribution of individual muscles. These patterns serve as target-specific references used during the projection-based assistance of myoelectric control.

To define the direction-specific reference muscle activation pattern $m_{ref}\left(\vartheta \right)$ , we first calculated $m_{med}\left(\vartheta \right)$ , the median muscle activation vector for each target direction ϑ across the healthy participants used to determine $H_{std}$ . Specifically, for each subject, the average EMG envelope across all repetitions of a given direction from target go until the target has been reached was calculated Then, the median vector across subjects of the direction-specific average activations was taken. This procedure provided a robust assessment of the typical activation pattern for each direction, capturing physiologically plausible features while reducing the influence of outliers or inter-subject variability. During stroke patients’ myoelectric control, muscle activations are translated into output forces via a linear mapping defined by the standardized EMG-to-force matrix ( $H_{std}$ , see 2.3). However, directly using $m_{med}\left(\vartheta \right)$ as input to this mapping does not ensure that the resulting force $H_{std}\cdot m_{med}\left(\vartheta \right)$ will align with the desired target. For this reason, we refined $m_{med}$ through a quadratic optimization procedure to obtain the final reference pattern $m_{ref}\left(\vartheta \right)$ . This optimization, implemented using the *quadprog* function in MATLAB, was subject to the constraints that the resulting force must exactly match the desired force to reach the target ( $f_{target}\left(\vartheta \right)=H_{std}\cdot m_{ref}\left(\vartheta \right)$ ) and that $m_{ref}$ must not have negative activations ( $m_{ref}\geq 0).$ Within these constraints, the optimization aimed at maximizing the similarity between $m_{ref}$ and $m_{med}$ to maintain the physiological meaning while minimizing the norm of $m_{ref}$ to reduce co-contraction and promote efficient muscle activation. The objective function used for this optimization was formulated as:

$$J\left(m\right)=m_{ref}^{T}\cdot m_{ref}-\lambda \cdot m_{ref}^{T}\cdot m_{med}$$

where λ is a weighting parameter that balances the trade-off between minimizing co-contraction and preserving physiological similarity to the median activation pattern. The selection criterion was designed to ensure a stable solution. To this end, we analyzed how the norm of $m_{ref}$ varied as a function of λ . The smallest value of λ at which further reductions led to changes smaller than $10^{-4}$ was chosen (Figure 4A). This approach identified an optimal λ value of $1.2\cdot 10^{-3}$ . At this value, the similarity between $m_{ref}$ and $m_{med}$ exceeded 0.95, ensuring that the optimized activation patterns maintained a high degree of physiological plausibility while effectively reducing co-contraction. Following this optimization process, the reference muscle patterns $m_{ref}$ were established for each target direction ϑ and their profiles are presented in Figure 4B.

Having defined $m_{ref}$ , the assisted muscle activation $m^{*}\left(\vartheta \right)$ for each target direction is determined as a weighted combination of the actual muscle pattern ( $\left.m\right)$ and the muscle pattern projected on the reference pattern $m_{proj}$ :

$$m^{*}\left(\vartheta \right)=\left(1-\alpha \right)\;m+\alpha \;m_{proj}\left(\vartheta \right)$$

with α being the level of assistance that is adjusted during training. At full assistance ( α=1 ), cursor movement aligns with the target direction, simulating optimal muscle activation. To promote active motor engagement, assistance is progressively reduced based on patient performance, ensuring a consistent level of task difficulty. As assistance decreases, deviations in cursor trajectory provide real-time feedback on the muscle activation adjustments required to achieve accurate control.

To ensure that the assisted muscle activation $m^{*}$ can effectively control the virtual cursor, the $H_{std}$ must be scaled to produce forces that allow to reach the target given the patient’s actual capabilities. To achieve this, an initial scaling procedure is applied based on the performance of the LAL, providing an individual measure of force generation capability in the context of the specific task. Before the first therapy session, data are collected from the LAL during isometric force control tasks, as described below in 2.6.1. Offline cursor trajectories are reconstructed using the $H_{std}$ and the $m_{LAL}^{*}\;\left(\vartheta \right)$ with total assistance ( $\left.\alpha =1\right)$ . For each target direction, the maximum amplitude of the reconstructed force ( $\left.{\left\|f_{LAL}^{*}\;\left(\vartheta \right)\right\|\;}_{\max}\right)$ is computed, and a scaling factor $\gamma \left(\vartheta \right)$ is determined by dividing the force magnitude corresponding to the target distance $\left(d=\left\|f_{target}\left(\vartheta \right)\right\|\right)$ by this value:

$$\gamma \left(\vartheta \right)=\frac{d}{{\left\|f_{LAL}^{*}\;\left(\vartheta \right)\right\|\;}_{\max}},\text{with }f_{LAL}^{*}\;\left(\vartheta \right)=H_{std}\;m_{LAL}^{*}\;\left(\vartheta \right)$$

This factor ensures that the reconstructed trajectory accurately reaches the target. To obtain a single global scaling factor for $H_{std}$ the average value of $\gamma \left(\vartheta \right)$ across all the target directions is taken, and the scaled $H_{std}$ will be:

$$H_{std}^{scaled}=\bar{\gamma }\;H_{std}$$

To account for direction-specific motor impairment of the MAL, an additional scaling factor is required. In stroke patients, impairment levels may vary across movement directions and training sessions, necessitating individualized adjustments. To compute this additional scaling parameter for the MAL, the same approach used to determine γ is applied. However, instead of using data from the LAL, trajectories are reconstructed from the three force control blocks performed by the patient in the session with the MAL, as described below in 2.6.2. This reconstruction is carried out using the $H_{std}^{scaled}$ and the $m_{MAL}^{*}\;\left(\vartheta \right)$ with total assistance $\left(\alpha =1\right)$ . Following the same procedure, the scaling parameter $\beta \left(\vartheta \right)$ is computed as:

$$\beta \left(\vartheta \right)=\frac{d}{{\left\|f_{MAL}^{*}\;\left(\vartheta \right)\right\|\;}_{\max}},\text{with }f_{MAL}^{*}\;\left(\vartheta \right)=H_{std}^{scaled}\;m_{MAL}^{*}\;\left(\vartheta \right)$$

A value of β=1 indicates that the scaling for the MAL is identical to that of the LAL, providing an additional measure to assess the patient’s motor performance across different movement directions and sessions. Indeed, the scaling parameter $\beta \left(\vartheta \right)$ is updated at each session to account for potential changes in patient performance. This approach also facilitates the monitoring of parameter variations across each target direction, providing an additional metric for assessing motor learning. Now, integrating all the components described above, the final force applied to control the virtual cursor is given by:

$$f\left(\vartheta \right)=\beta \left(\vartheta \right){\;H}_{std}^{scaled}\;m^{*}\left(\vartheta \right)$$

### 2.6 Therapy session protocol for stroke patients

Each session has a maximum duration of 30 minutes, balancing therapeutic effectiveness with minimization of the patient’s fatigue. Each session consists of a series of blocks of eight trials, with each trial corresponding to one of the eight target directions spaced at 45° intervals.

#### 2.6.1 Least Affected Limb (LAL) session

The LAL session begins with a block of Maximum Voluntary Contraction (MVC) trials, which are used to normalize the force required to reach the targets in the following sessions, i.e., the target distance *d*, to 20% of the average force recorded during this block. During MVC trials, patients are asked to exert the maximum isometric force in each of the eight directions in the task space. Then, the patient performs six blocks of FC reaching, totaling 48 trials. This session serves two main purposes: establishing the scaling parameters required by the assistance algorithm during the therapy session (see 2.5) and allowing the patient to familiarize themselves with the task.

#### 2.6.2 Most Affected Limb (MAL) session

Each therapy session consists of a sequence of myoelectric control tasks utilizing the assistive-adaptive algorithm described in Section 2.5. As in the LAL session, therapy with the MAL starts with a block of MVC trials to normalize the force required for target-reaching to 20% of the MVC force. Additionally, a set of three FC blocks follows this initial normalization phase. Then, two pre-assistance myoelectric control blocks (16 trials in total, corresponding to two repetitions for each of the eight target directions), during which the assistance level is set to α=0 , are performed. These blocks assess the patient’s baseline performance without external assistance. After this initial assessment, a fully assisted block ( α=1 ) is conducted to evaluate the patient’s ability to reach the targets under maximum assistance conditions. The assistance level in subsequent blocks is then adjusted according to the performance in the preceding block. If the patient successfully reaches at least seven out of eight targets, the assistance level is reduced decreasing α by 0.1 in the following block. If the patient fails to reach at least seven targets, the block is repeated with the same assistance level. This procedure ensures a progressive reduction of assistance, encouraging active engagement and motor adaptation while preventing excessive difficulty that could discourage the patient. At the end of each session, after 25 min of therapy, two additional post-assistance myoelectric control blocks (16 trials in total, two per direction) are performed without assistance ( α=0 ), allowing for an assessment of improvements in motor control throughout the session.

### 2.7 Data analysis

Offline reconstruction of trajectories using $H_{std}$ *:* To investigate the feasibility of using the standardized mapping matrix $H_{std}$ , we performed offline trajectory reconstructions on data previously collected from healthy participants. EMG data recorded during the myoelectric control block were reprocessed using $H_{std}$ . Specifically, the vectors of EMG signals were multiplied by the standardized matrix to generate estimated force vectors, which were then passed through the same adaptive mass-spring-damper (MSD) filter used during real-time control. This allowed us to reconstruct the cursor trajectory that would have been generated as the subject used the standard matrix during the task.

Similarity Between Muscle Activation Patterns: To assess the effectiveness of the assistive-adaptive algorithm, we computed the similarity between the average filtered muscle activation pattern recorded during each trial and the reference activation pattern used for projection. As a measure of similarity, we computed the scalar product between the two muscle activation patterns. This measure indicates how closely the patient’s muscle activity aligns with the optimized reference pattern over the session. Higher similarity values indicate a successful adaptation to the reference functional muscle space.

Initial Direction Error: The Initial Direction Error (IDE) quantifies the angular deviation between a straight-line trajectory toward the target and the actual movement direction initiated by the participant. Specifically, the IDE is computed as the absolute angle between the target direction and the instantaneous trajectory vector measured 0.1 s after the target onset (target go). This metric provides a sensitive assessment of the accuracy of initial motor planning and the participant’s ability to generate a correct feedforward command. Lower IDE values reflect more accurate movement initiation, indicating improved motor planning and reduced neuromuscular deficits during the early phase of trajectory execution in myoelectric control.

Path Length: The Path Length (PL) quantifies the total distance traveled by the cursor during each trial and serves as a measure of movement efficiency. It is computed as the sum of the Euclidean distances between consecutive points along the cursor trajectory from movement onset to target acquisition. Formally, if the cursor trajectory is sampled at n time points with positions $x_{1},x_{2},\ldots ,x_{N}$ , the path length is calculated as:

$$PL=\sum_{i=1}^{n-1}\left\|x_{i+1}-x_{i}\right\|$$

Shorter path lengths indicate more direct and controlled movements toward the target, while longer paths suggest deviations, corrections, or hesitations. A reduction in PL over time is typically interpreted as a sign of improved motor coordination and growing familiarity with the myoelectric control system.

#### 2.7.1 Statistical analysis

To evaluate the effect of the assistive-adaptive algorithm on performance, a linear mixed-effects model was employed. The experimental condition (pre vs. post intervention) was modeled as a fixed effect, while the direction of the target was included as a random effect to account for variability across conditions without testing it explicitly, given the limited number of repetitions. Statistical significance was determined by examining the fixed effect associated with the condition; differences were considered significant at p < 0.05.

## 3 Results

### 3.1 Offline reconstructed trajectories in healthy participants demonstrate validity of $H_{std}$

**FIGURE 5:**
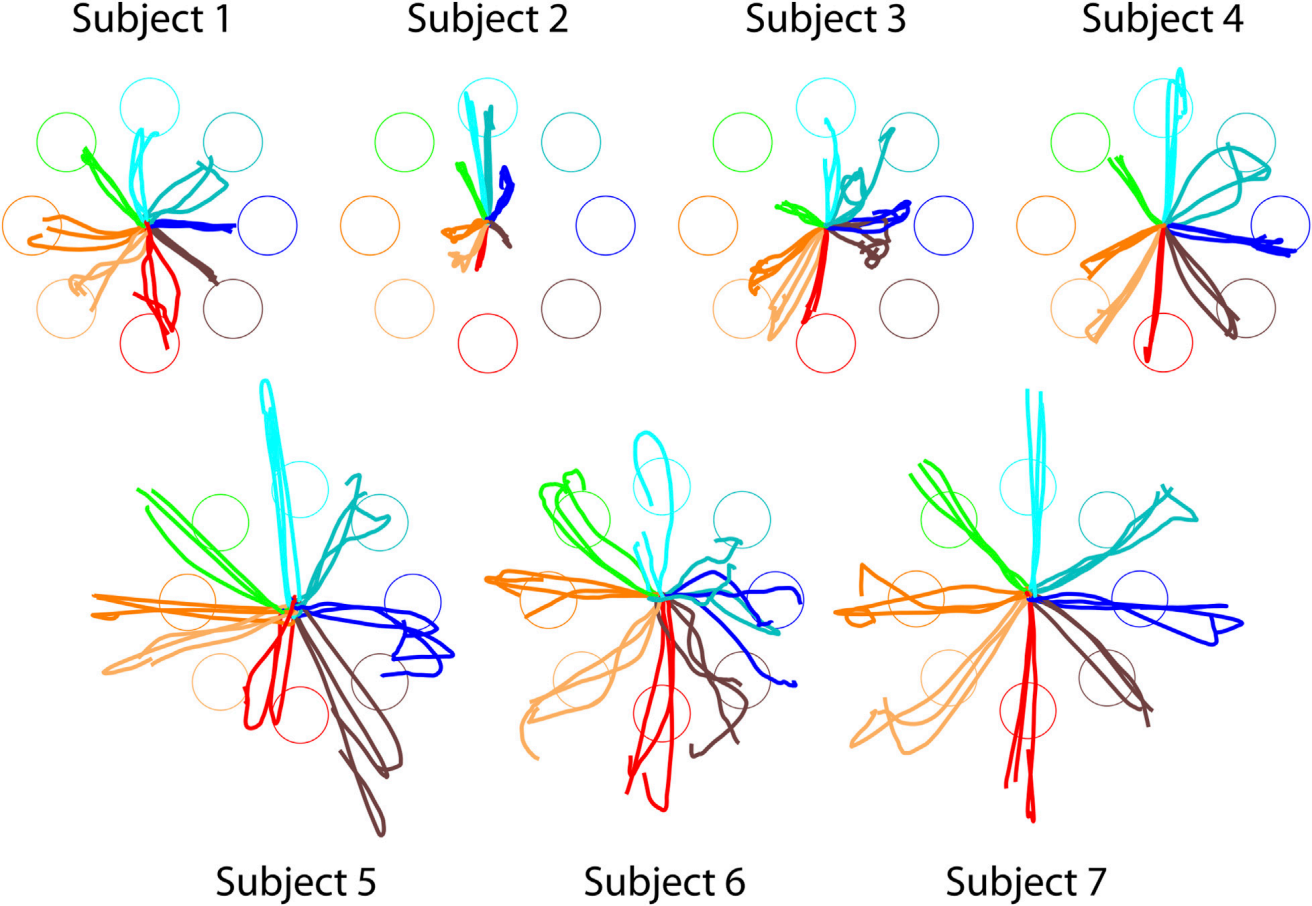
Offline reconstructed cursor trajectories of healthy participants using standardized EMG-to-force mapping ( $H_{std\;}$ ). Each trajectory is color-coded according to the corresponding target. Trajectories are displayed from the “go” signal ltarget acquisition.

Initially, we tested the mapping between muscle activity and applied forces using $H_{std}$ in healthy participants. The offline-reconstructed trajectories (Figure 5) were directed towards the targets with a good directional accuracy (20.4° ± 15.3° IDE, mean ± SD, averaged across subjects), suggesting that $H_{std}$ preserves the general structure of the myoelectric control. For comparison, the same subjects achieved an average IDE of 8.02° ± 2.09° (mean ± SD, averaged across subjects) during online myoelectric control with their own subject-specific mappings. Although less precise, the performance with $H_{std}$ still demonstrates that the proposed standardized approach can support functional cursor control. It is important to note that these trajectories were reconstructed in post-processing and do not reflect the actual movements of the cursor during the original sessions. Nonetheless, we hypothesize that in a real-time scenario, the presence of visual feedback could support subjects in further refining their motor output when using $H_{std}$ , thereby improving accuracy and making the control physiologically meaningful. In addition, discrepancies in trajectory amplitude were observed, highlighting the need for subject-specific scaling to ensure accurate force magnitude representation.

### 3.2 Improvement in movement quality in stroke patients demonstrates the potential of patient-tailored assistance

**FIGURE 6:**
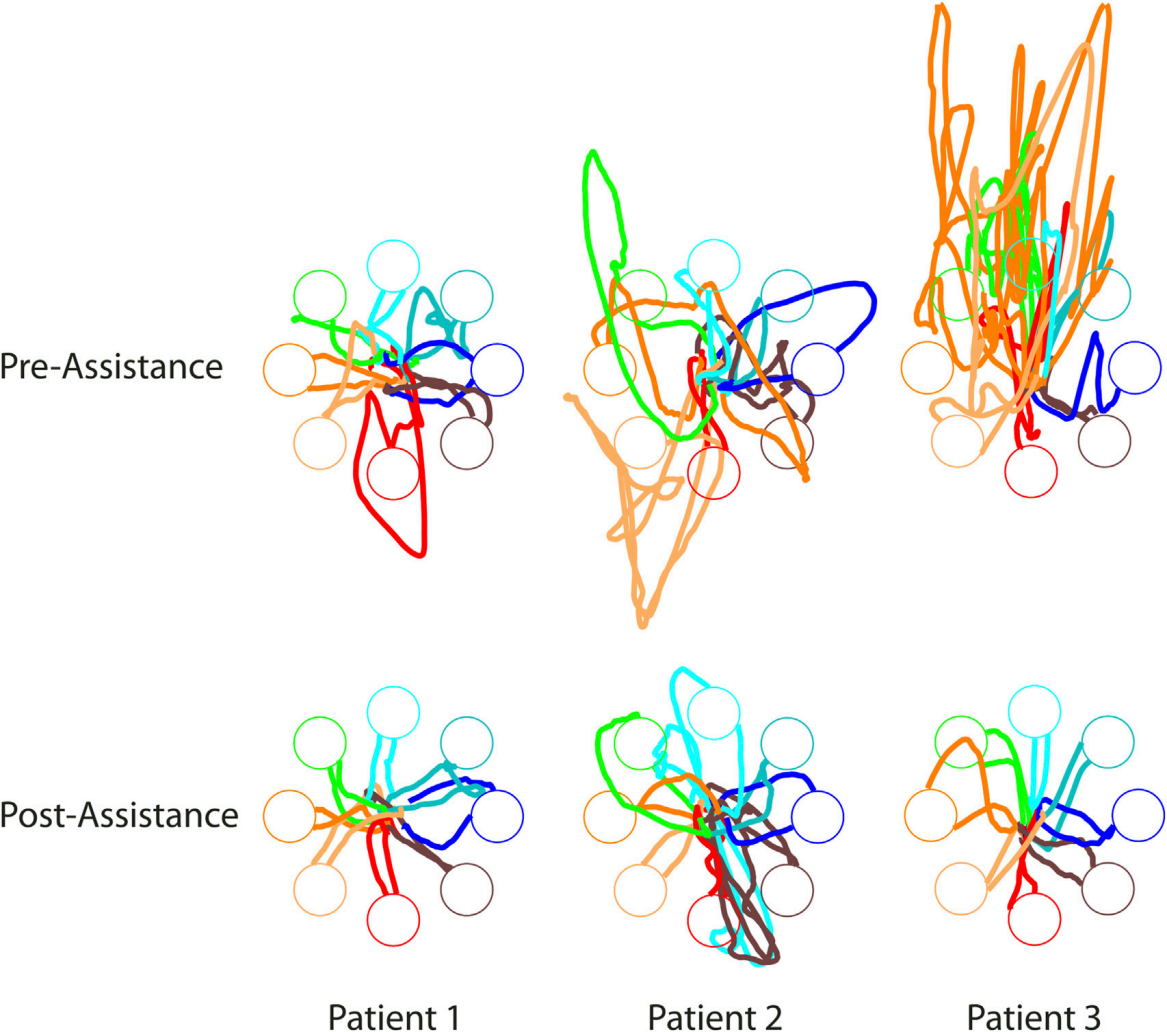
Cursor trajectories of the three stroke survivors, before (top) and after the patient-tailored assistance. Each trajectory is color-coded according to the corresponding target. Trajectories are displayed from the “go” signal to target acquisition.

Three stroke survivors participated in the study using the protocol outlined in 2.6. These pilot experiments consist of a single session and therefore do not provide a clear indication of the efficacy of therapy over multiple sessions. However, they do provide a preliminary assessment of the feasibility of the assistive-adaptive algorithm for assistive myoelectric control in stroke patients. All three stroke patients showed improved kinematic performances following the patient-tailored assistance (Figure 6), which will be explained in detail in the following subsections.

#### 3.2.1 Motor adaptation following assistive training

**FIGURE 7:**
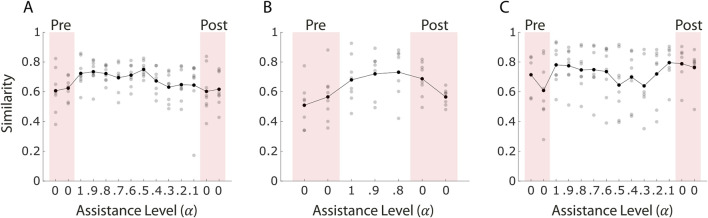
Similarity between recorded muscle activity and functional reference patterns across blocks for the three stroke survivors **(A–C)**. Similarity values range from 0 to 1, with one indicating perfect alignment between the two muscle patterns. Each grey dot represents the similarity value of an individual trial, while black dots indicate the average similarity within each block. Red-shaded areas correspond to pre- and post-assistance blocks (α = 0), during which no projection-based assistance was provided. White areas represent assisted blocks. The figure illustrates how muscle activation aligned with the target-specific reference patterns throughout the session.

Patient 1 (Figure 7A) showed similar values between the pre- and post-assistance blocks, suggesting limited short-term adaptation in terms of similarity. The similarity values, averaged across trials, were 0.61 and 0.62 in the two pre-assistance blocks, and 0.60 and 0.62 in the two post-assistance blocks. No statistically significant difference was found between pre- and post-assistance values (p = 0.750). However, during the assisted blocks, a transient increase in similarity was observed at higher levels of assistance. In the first assisted block ( α=1 ), the similarity reached 0.72. As the level of assistance was progressively reduced, the similarity decreased reaching a value of 0.64 in the final assisted block. These findings suggest that while the projection helped reinforce functional activation pattern under assistance, the patient had struggled maintaining alignment as the control became more active.

Patient 2 (Figure 7B) exhibited a significant difference in similarity values between the pre- and post-assistance blocks (p = 0.044), with an increase especially observed in the first post-assistance block. Averaged similarity values were 0.51 and 0.57 during the pre-assistance phase, increasing to 0.69 in the first post-assistance block before decreasing to 0.57 in the second one, returning to baseline levels. This patient completed only three assisted blocks ( α=1,0.9,0.8 ) due to the 30-min session limit. During these assisted blocks, similarity increased, peaking at 0.73 in the final assisted trial.

Patient 3 (Figure 7C) demonstrated a clearer and more consistent pattern of improvement. A highly significant increase in similarity was found between the pre- and post-assistance blocks (p < $10^{-3}$ ), with average values rising from 0.71 to 0.61 (pre-assistance) to 0.79 and 0.76 (post-assistance). During the assisted phase, similarity initially increased to 0.78 in the first block ( α=1 ), then declined with lower assistance values, reaching 0.64 at α = 0.3. Notably, in the final two assisted blocks (α = 0.2 and 0.1), similarity increased again, peaking at 0.80 in the final assisted block. This suggests that the patient was able to learn and progressively align their muscle activations with the reference pattern as the control became more active, maintaining this adaptation even after assistance was removed.

#### 3.2.2 Motor planning after assistive training

**FIGURE 8:**
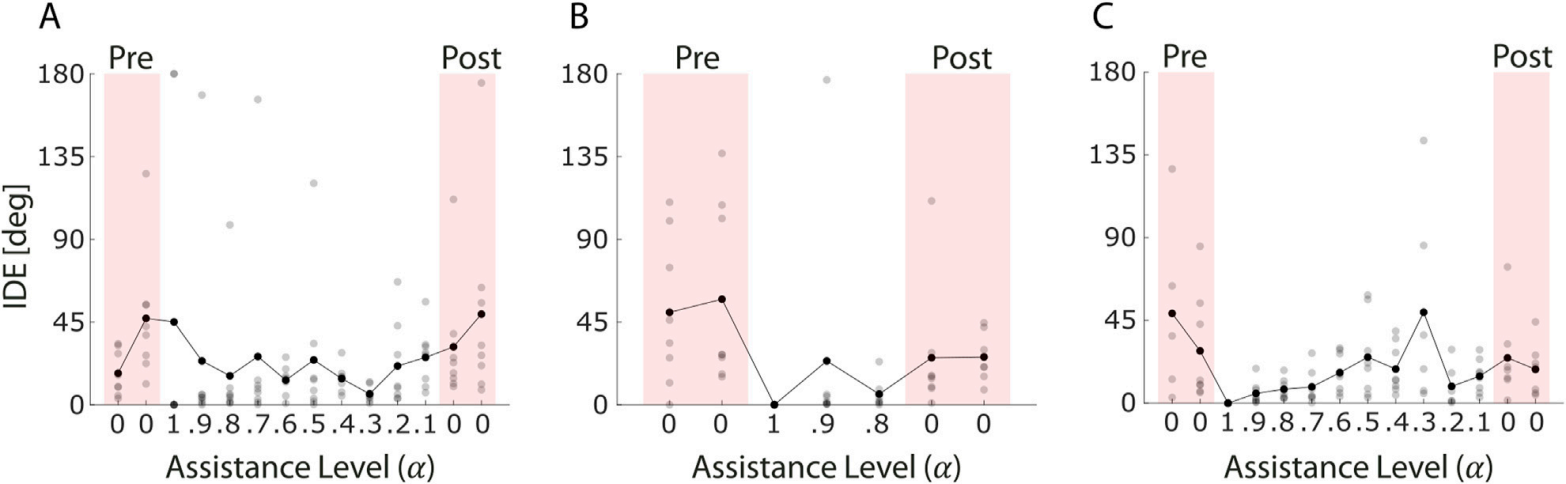
Initial Direction Error (IDE) across blocks for the three stroke survivors **(A–C)**. Each grey dot represents the IDE value of an individual trial, while black dots indicate the average IDE within each block. Red-shaded areas correspond to pre- and post-assistance blocks (α = 0), during which no projection-based assistance was provided. White areas represent assisted blocks. The figure shows how movement initiation accuracy evolved throughout the session.

Patient 1 (Figure 8A) showed no significant change in IDE between pre- and post-assistance blocks (p = 0.53). The average values were highly variable across blocks but within similar ranges (pre-assistance: 17.2° and 47.0°; post-assistance: 31.5° and 49.3°), suggesting that a single session of myoelectric control with adaptive assistance was not sufficient to influence the patient’s initial movement planning.

In contrast, Patient 2 (Figure 8B) exhibited a significant reduction in IDE values following the assistance phase (p = 0.019). Despite completing only three blocks of assisted control, limited by the 30-min session duration, the patient improved initial directional accuracy during post-assistance trials. This result suggests that even a relatively brief exposure to the assistive-adaptive algorithm may support short-term improvements in movement planning for some individuals.

Patient 3 (Figure 8C), on the other hand, showed a decrease in average IDE values between pre- and post-assistance blocks (pre-assistance: 49.8° and 29.4°; post-assistance: 23.6° and 13.2°), even though the difference was not statistically significant (p = 0.17). Nevertheless, the trend may indicate a partial refinement in the initial motor trajectory.

#### 3.2.3 Movement efficiency following assistive training

**FIGURE 9:**
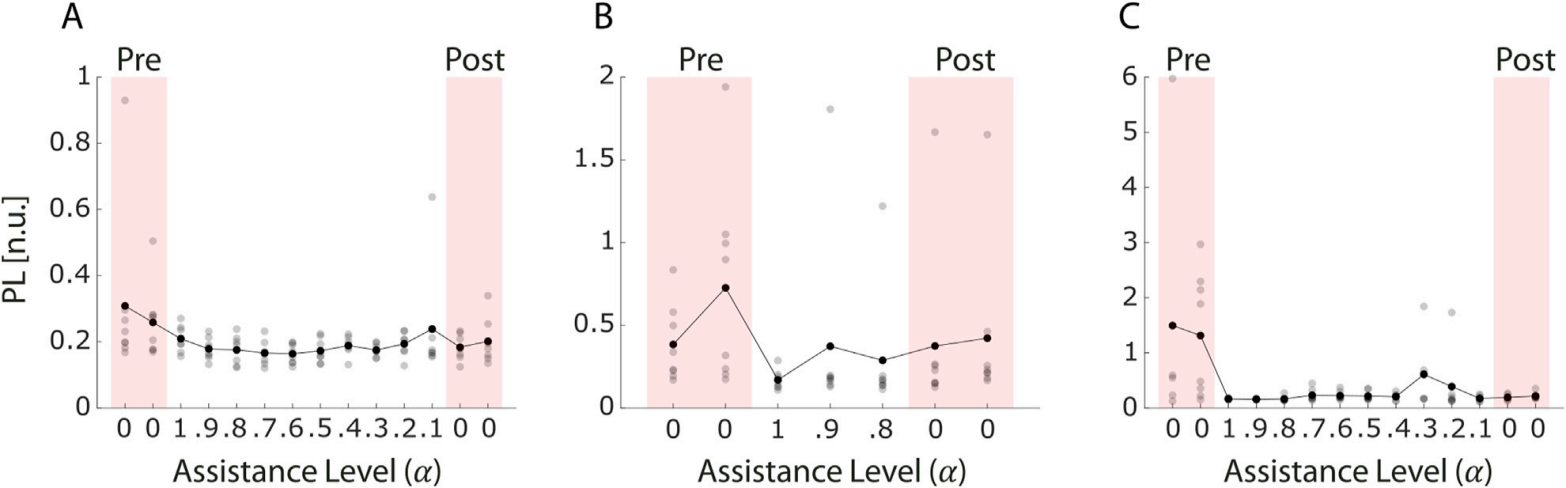
Path Length (PL) across blocks for the three stroke survivors **(A–C)**. PL values were normalized to the average force recorded during the maximum voluntary contraction (MVC) block, in which each participant was asked to exert maximal isometric force in all eight task directions. Each grey dot represents the PL value of an individual trial, while black dots indicate the average PL within each block. Red-shaded areas correspond to pre- and post-assistance blocks (α = 0), during which no projection-based assistance was provided. White areas represent assisted blocks. This figure reflects changes in movement efficiency and trajectory optimization during the session.

Patient 1 (Figure 9A) showed a reduction in average PL values from pre-to post-assistance blocks (pre-assistance: 0.31 and 0.27; post-assistance: 0.18 and 0.20), even though this trend did not reach statistical significance (p = 0.053).

In Patient 2 (Figure 9B), despite significant improvements in similarity and IDE, no significant change was observed in PL (p = 0.36). This suggests that although the patient may have improved in terms of muscle coordination and movement planning, the accuracy or efficiency in reaching the target was not yet optimized.

Patient 3 (Figure 9C), in contrast, showed a significant reduction in PL between pre- and post-assistance phases (p = 0.004), with average values decreasing from 1.49 to 1.31 (pre-assistance) to 0.19 and 0.21 (post-assistance). Combined with the increased similarity to reference patterns, these results indicate substantial improvements in both muscle coordination and task execution efficiency for this patient.

### 3.3 Anticipated results

The preliminary data collected from three pilot cases offer encouraging yet limited insights into the short-term effects of the proposed assistive-adaptive control strategy. Each patient completed a single therapy session, during which we observed modest but consistent trends suggesting that the algorithm may support improved motor execution and coordination within the boundaries of a single exposure. Building on these initial observations and the theoretical hypothesis underlying the algorithm, we anticipate that repeated exposure to the training protocol will lead to progressive and more stable improvements. Across multiple training sessions, the combination of adaptive assistance, real-time feedback, and the use of a standardized functional mapping is expected to reinforce functional activation patterns, reduce pathological co-contractions, and enhance voluntary motor control. Patients are expected to progressively internalize more effective muscle strategies, improving both, movement quality and autonomy. We expect these neuromuscular improvements to translate into clinically meaningful functional gains. One of the primary anticipated outcomes is a significant increase in scores on the Fugl-Meyer Assessment for the Upper Extremity (FMA-UE), reflecting enhanced motor capacity and coordination of the MAL. A sustained improvement in FMA-UE would provide strong evidence that the proposed strategy is not only effective at guiding motor performance during the task but also capable of driving broader functional recovery. In summary, while these pilot data support the system’s capacity to induce short-term motor benefits, we expect a full multi-session therapy protocol to reinforce these effects over time, enabling durable neuroplastic changes and significant clinical improvements.

## 4 Discussion

This study introduces an assistive-adaptive algorithm for myoelectric control in stroke rehabilitation, designed to explore its potential for promoting the re-learning of functional muscle activation patterns. We hypothesize that enhanced motor recovery can be achieved using physiologically meaningful visual feedback delivered in virtual reality (VR). The proposed approach enables patients to perform isometric reaching tasks using the muscle activity recorded in real-time from their MAL. These signals are processed through a standardized, functional EMG-to-force mapping derived from healthy individuals, ensuring that motor output reflects typical coordination patterns rather than post-stroke abnormalities.

A projection mechanism transforms the impaired muscle activity into more functional motor outputs, correcting the muscle activity in a physiologically plausible movement direction while preserving the patient’s active participation. In healthy individuals, myoelectric control based on physiological mapping occurs quite naturally, as demonstrated in our previous studies (e.g., see Berger and d’Avella, 2023a; Berger and d’Avella, 2023b). However, for stroke survivors, this type of control can be challenging because of their dysfunctional muscle activity, often marked by high levels of muscle co-activation (Dewald et al., 1995; Cheung et al., 2012; Seo et al., 2022), along with muscle weakness and spasticity (Beer et al., 2000; Zackowski, 2004).

In recent years, several research groups have started exploring the use of myoelectric control to support motor recovery after stroke. Many approaches rely either on EMG signals from the LAL, applying mirroring strategies (Sarasola-Sanz et al., 2018), or on activating isolated muscle pairs, limiting control on specific movement directions and a non-physiologically mapping between force and muscle activations (Mugler et al., 2019; Seo et al., 2022). Although such methods show potential, to date mirrored EMG control, i.e., from the LAL, often results in poor performance when applied to stroke survivors, while pairwise muscle training typically leads to simplified mappings that do not reflect the natural relationship between muscle activity and force direction.

The above-mentioned limitations highlight the need for solutions that support voluntary control on the MAL, especially in patients with severely impaired motor output. Our approach addresses these limitations by operating directly on the MAL and translating muscle activity into cursor movements using a standardized mapping derived from healthy individuals. By using this functional mapping to promote motor re-learning, our system avoids reinforcing dysfunctional activation patterns. This is crucial because patient-specific mappings based on impaired signals are unlikely to provide a meaningful mapping for myoelectric control. Instead, using a healthy reference allows patients to interact with the system as if their muscle activations were less dysfunctional, creating conditions that may favor motor re-learning. An alternative to using a standardized mapping derived from healthy individuals could be to use the mapping estimated from the LAL of each patient. While this approach might appear intuitive, since it relies on the patient’s own functional muscle activations, it comes with several important limitations. Evidence suggests that even the LAL may not exhibit fully functional control, especially in cases of bilateral motor deficits or compensatory strategies that alter natural muscle coordination (Nakayma et al., 1994; Kitsos et al., 2013). Moreover, this strategy would eliminate the advantage of using a standardized reference, since each patient would effectively have a unique control scheme. This lack of consistency would make it difficult to compare performance across individuals and to generalize findings. We thus hypothesize that a standardized, healthy reference mapping provides a consistent and physiologically grounded baseline that can promote motor re-learning while enabling systematic evaluation and comparison of results across patients.

The integration of real-time visual feedback, a well-established driver of motor learning (Krakauer et al., 2019), may help patients to associate the relationship between their activations and the resulting movement, reinforcing positive adaptations. The projection mechanism not only corrects movement trajectories but also allows patients to complete tasks successfully even when EMG signals are dysfunctional, supporting engagement and motivation during the therapy. Over time, the level of assistance can be gradually reduced, encouraging voluntary and active muscle control.

The analysis of the three pilot sessions revealed individual variability in the short-term response to the assistive-adaptive myoelectric control system. In terms of similarity between muscle activations and the reference pattern, Patient three showed the most robust response, with a statistically significant increase maintained after the removal of assistance. This supports the potential for the projection mechanism to facilitate the alignment of muscle activity with functional patterns, at least in participants capable of adapting within a single session. Patient two also showed a significant increase in similarity, even though this effect was not maintained in the second post-assistance block. In contrast, Patient one did not exhibit any significant changes, and only transient increases were observed during blocks with high assistance. These findings highlight that while the system can temporarily enhance the use of functional muscle patterns, sustained improvements may not occur uniformly and may depend on the patient’s baseline motor condition or adaptability. In terms of IDE, significant improvements were observed only in Patient 2 after the assisted blocks, despite completing only three of them. This finding indicates that even a limited exposure to the assistive algorithm can improve motor planning. Notably, in this patient, the reduction in IDE occurred alongside the increase in muscle pattern similarity with the functional reference, suggesting a potential link between improved coordination at the muscular level and enhanced feedforward control. Patient three also showed a decrease in IDE values, although not statistically significant, while Patient one exhibited no meaningful change. Regarding PL, all three patients showed reductions of average values in post-assistance blocks, with only Patient three displaying a statistically significant improvement. For this patient, reductions in PL were accompanied by significant increases in similarity and, although not statistically significant, by a reduction in average IDE values after the assisted blocks. These results indicate a possible link between improved muscle coordination and more efficient movement planning and execution.

Overall, these pilot results indicate that the proposed assistive-adaptive algorithm can produce measurable short-term benefits in selected aspects of motor control, particularly when improvements across multiple metrics converge. However, the variability in the results across participants, combined with the need to understand whether some of the observed short-term improvements can be retained and consolidated over time, highlights the need for longitudinal interventions.

Existing evidence showed that neuroplastic changes and adaptations typically require large numbers of task repetitions (Lang et al., 2009; Kimberley et al., 2010). However, stroke patients, particularly those with severe impairments, may not tolerate long or highly demanding sessions. For this reason, we hypothesize that meaningful motor improvements and neural reorganization are more likely to emerge through repeated exposure to the algorithm across multiple, closely spaced sessions. This hypothesis is supported by a study (Nocilli et al., 2024) in which the authors found that the synergy structure of stroke patients gradually aligned with that of the LAL after several sessions of isometric reaching. Based on this evidence, a longer intervention involving a structured longitudinal protocol will be essential to assess whether the short-term effects observed in a single session can accumulate and translate into sustained and clinically relevant improvements in motor function.

Despite these promising outcomes, several limitations must be acknowledged. The findings are based on a small sample (n = 3). Thus, this limited sample size does not allow us to evaluate the variability across individuals. The present results should therefore be viewed as a proof-of-concept of the feasibility of the proposed approach, rather than as a test of clinical efficacy. Additionally, while patient sessions were conducted in fully immersive VR with a Meta Quest 3S headset, the healthy participants who provided the reference mapping ( $\left.H_{std}\right)$ were trained on a standard monitor. This mismatch in visual feedback may have shaped their motor behavior and could limit how well the healthy mapping generalizes to VR-based therapy. Furthermore, there was a considerable age difference between the stroke patients and healthy participants, which could have influenced muscle activation patterns. Nevertheless, the $H_{std}$ was intended to provide a general physiological reference rather than a perfectly age-matched control.

The performance of EMG-control is inherently sensitive to the quality of signal acquisition and processing. While efforts were made to standardize electrode placement and apply appropriate filtering, issues such as muscle cross-talk, movement artifacts, power line interference, and variable electrode-skin contact can introduce noise and affect both the accuracy of the estimated force and the stability of control. These aspects are particularly relevant in stroke populations, where spasticity or fatigue may further degrade signal quality (Zhang et al., 2013).

Finally, while our assistive control paradigm assumes that projecting onto a healthy activation manifold and gradually decreasing the assistance will guide them toward more functional motor patterns, this remains to be validated through long-term, controlled interventions. This study focuses on isometric tasks, which, although controlled and reproducible, do not fully capture the complexity of dynamic, functional movements encountered in daily life. Future work should explore the extension of this approach to dynamic reaching or task-oriented activities to evaluate its generalizability as well as its applicability to different pathologies, such as, e.g., cerebral palsy.

In conclusion, this study presents a novel, assistive-adaptive myoelectric control algorithm that leverages a physiologically grounded EMG-to-force mapping and immersive VR feedback to promote functional motor re-learning in stroke survivors. By providing personalized assistance and enforcing healthy muscle activation patterns, the system supports active engagement and neural recovery. While further studies are needed to verify these findings and confirm the effectiveness of the proposed approach, the results offer a promising advancement in post-stroke rehabilitation technologies.

## Data Availability

The raw data supporting the conclusions of this article will be made available by the authors, without undue reservation.
